# Low Cloud Cover-Adjusted Ultraviolet B Irradiance Is Associated with High Incidence Rates of Leukemia: Study of 172 Countries

**DOI:** 10.1371/journal.pone.0144308

**Published:** 2015-12-04

**Authors:** Raphael E. Cuomo, Cedric F. Garland, Edward D. Gorham, Sharif B. Mohr

**Affiliations:** 1 Division of Global Health, Department of Family Medicine and Public Health, University of California San Diego, La Jolla, California, United States of America; 2 Graduate School of Public Health, San Diego State University, San Diego, California, United States of America; 3 Division of Epidemiology, Department of Family Medicine and Public Health, University of California San Diego, La Jolla, California, United States of America; Van Andel Institute, UNITED STATES

## Abstract

There are 52,380 cases of leukemia and 24,090 deaths from it in the US annually. Its causes are unknown and no preventive strategies have been implemented. We hypothesized that leukemia is due mainly to vitamin D deficiency, which is due mainly to low solar ultraviolet B (UVB) irradiance. To test this hypothesis, we estimated age-standardized cloud-cover-adjusted winter UVB irradiance using cloud cover data from the International Satellite Cloud Climatology Project, latitudes of population centroids, and standard astronomical calculations. Incidence rates for 172 countries, available from the International Agency for Cancer Research, were plotted according to cloud-adjusted UVB irradiance. We used multiple regression to account for national differences in elevation and average life expectancy. Leukemia incidence rates were inversely associated with cloud-adjusted UVB irradiance in males (*p* ≤ 0.01) and females (*p* ≤ 0.01) in both hemispheres. There were few departures from the trend line, which was parabolic when plotted with the equator at the center of the display, northern hemisphere countries on the right side and southern hemisphere countries on the left. The bivariate association displayed by the polynomial trend line indicated that populations at higher latitudes had at least two times the risk of leukemia compared to equatorial populations. The association persisted in males (*p* ≤ 0.05) and females (*p* ≤ 0.01) after controlling for elevation and life expectancy. Incidence rates of leukemia were inversely associated with solar UVB irradiance. It is plausible that the association is due to vitamin D deficiency. This would be consistent with laboratory studies and a previous epidemiological study. Consideration should be given to prudent use of vitamin D for prevention of leukemia.

## Introduction

It was estimated that the United States had 52,380 new cases and 24,090 deaths from leukemia in 2014 [1]. Globally, prevalence was estimated to be 352,000 cases in 2012 [2].

The four main subtypes of leukemia are chronic lymphocytic leukemia (CLL), acute lymphocytic leukemia (ALL), chronic myeloid leukemia (CML), and acute myeloid leukemia (AML). Numerous laboratory studies have been conducted on the HL60 line of AML cells [3–6]. These studies have supported a biological mechanism of action whereby leukemia cells are acted upon by vitamin D metabolites. Levels of vitamin D can be increased either by consumption or increased exposure to ultraviolet B (UVB). 1,25-Dihydroxyvitamin D has been found to aid myeloid leukemia differentiation to macrophages and monocytes [3]. One study found that 1,25-dihydroxyvitamin D resulted in differentiation by inhibiting the ERK5 pathway, thereby resulting in inhibition of G_1_ and G_2_ phases of the mitotic cycle [6]. Eleven of twelve leukemic mice administered long-term 1,25-dihydroxyvitamin D were found to have undetectable tumors after three years [5]. A separate study also found that supplementation with 1,25-dihydroxyvitamin D increased survival time in mice with myeloid leukemia [4]. In humans, a clinical study found that lower 25-hydroxyvitamin D levels was associated with poorer relapse-free survival, a finding that persisted even after controlling for smoking and white blood cell count [7]. A case-control study found that all patients with acute leukemia had 25-hydroxyvitamin D levels below 20 ng/ml [8].

Though a number of biological and epidemiological studies have been conducted upon AML, the biological mechanisms associated with vitamin D metabolites support an even greater preventive potential for CLL. Vitamin D metabolites have been shown to up-regulate epithelial cadherins, thereby stimulating contact inhibition of cancer, a well-known anticarcinogenic biological mechanism [9]. CLL commonly develops in older age groups, purportedly after several years of exposure to behaviors or environments that increase risk. These public health risk factors facilitate a cellular environment that stimulates cellular proliferation, to which cells are more resilient when higher levels of vitamin D have encouraged stronger contact inhibition. Therefore, vitamin D sufficiency may plausibly inhibit the development of CLL. Since CLL constitutes a relatively large proportion of total leukemia incidence [10], epidemiological analyses that assess relationships with total leukemia incidence may be largely explaining variability from CLL, to which there exists biological plausibility for an association with vitamin D status, in addition to AML, to which there exists a preponderance of published biological evidence for an association with vitamin D status.

There are several known risk factors for leukemia, including exposure to ionizing radiation, exposure to organic solvents, and having Down syndrome [11]. While robust data on several population-level risk factors may not exist, country-level data on certain characteristics that may confound the relationship between UVB exposure and leukemia incidence are available. Importantly, these include elevation, which may influence exposure to UVB, and life expectancy, which can influence risk for developing leukemia.

This study seeks to determine whether the global relationship between UVB exposure and leukemia incidence is consistent with laboratory and clinical findings of anticarcinogenic effects from higher levels of vitamin D. The main differences between this and a previous study [12] are that in this paper the graphic analyses are based on cloud-adjusted UVB irradiance, while in the former paper the only graphics were for incidence rates by latitude; and this report is of data for 2012, a decade later than the data that were used in the previous research. The newer method of analysis, which took cloud cover into account, yielded a substantially superior model.

## Materials and Methods

### Data Sources

Age standardized rates of leukemia were obtained for males and females separately from the International Agency for Cancer Research’s (IARC) Global Cancer (GLOBOCAN) 2012 database [13]. These incidence rates of leukemia were determined by IARC for each country using the best sources of surveillance data available. The source of data for total population for 2012 and sex-specific life expectancy for 2012 was the World Health Organization [14], and data for country-specific average elevation above sea level were obtained from Portland State University [15].

Cloud cover-adjusted UVB was estimated for each country from cloud cover data, available from the National Aeronautics and Space Administration’s (NASA) International Satellite Cloud Climatology Project (ISCCP) satellite [16], and from total noon solar irradiance at the winter solstice at the latitude of the population centroid of each country. Wintertime UVB was used because it corresponds to populations whose vitamin D photosynthesis is lowest, thereby corresponding to the development of cancer in those populations. Total noon solar irradiance was calculated from data on solar irradiance and geographic variations, available from NASA [17], and from latitude of population centroid, available from Columbia University’s Center for International Earth Science Information Network [18]. The formula used to obtain total noon solar irradiance at the top of the atmosphere on the date of the winter solstice in the northern hemisphere is A' = A * *cos* (x + 23.5 degrees), and the formula used to obtain these data for countries in the southern hemisphere is A' = A * *cos* (x—23.5 degrees). In these formulas, A’ is total country-specific solar irradiation (W/m^2^), A is total equatorial solar irradiation (W/m^2^), and x is latitude of the population centroid of each country. Total solar irradiance was then multiplied by 0.004, because UVB has been estimated as 0.4% of total solar irradiance. These values were then multiplied by (1 –fractional cloud cover) in order to adjust for cloud cover.

### Statistical Methods

**Fig 1:**
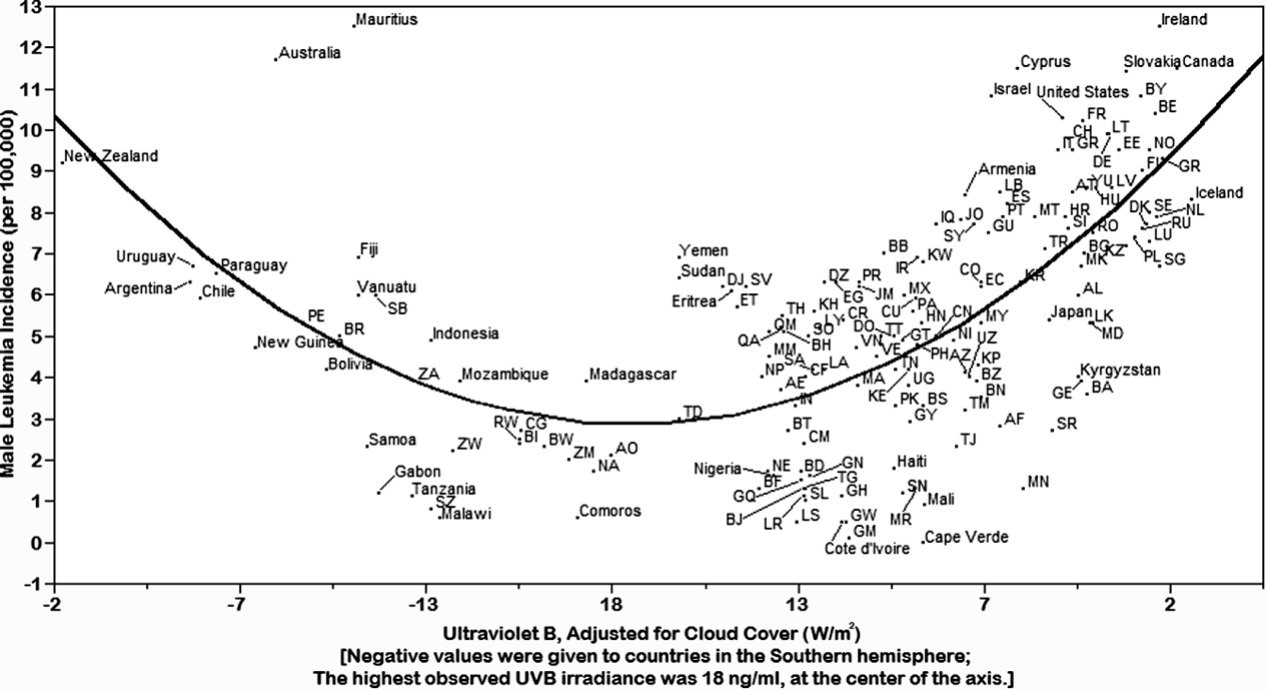
Rates of age-standardized leukemia incidence (cases per 100,000) in males according to cloud cover-adjusted UVB irradiance (W/m^2^), 172 countries, 2012.

**Fig 2:**
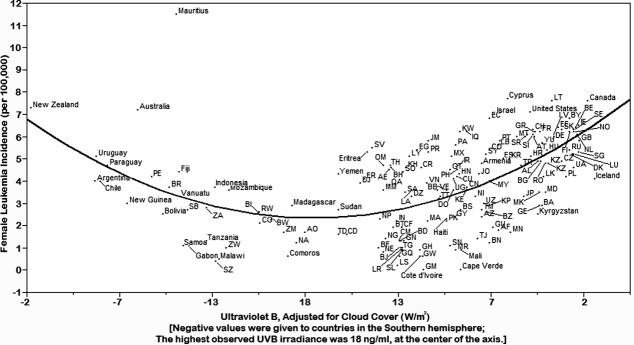
Rates of age-standardized leukemia incidence (cases per 100,000) in females according to cloud cover-adjusted UVB irradiance (W/m2), 172 countries, 2012.

Through an ecologic study design, SAS software (Cary NC: SAS Institute) was used to analyze data using multiple linear regression. A correction matrix of Pearson’s correlation coefficients was constructed with all variables included in regression analyses (Table 1). JMP software (Cary NC: SAS Institute) was used to plot incidence rates of sex-specific country-level leukemia against cloud cover-adjusted UVB with a polynomial trend line (Fig 1, Fig 2). A graph was also constructed that displays the relationship between total country-level leukemia incidence and cloud cover-adjusted UVB while weighting for total country population in 2012 (S1 Fig). To best display a potential equatorial association with cancer incidence, negative values were given to countries in the southern hemisphere. For the main sex-specific graphs, points were labeled with two-letter country codes, and, when space permitted, full country labels were entered. ArcGIS v10.2 (Redlands CA: Esri) was used to produce a world map that displays graduated symbols for leukemia incidence in both sexes, on top of a choropleth gradient representing cloud cover-adjusted UVB for each country (S2 Fig). Though the correlation table and figures include nearly all countries (n = 172), the involvement of additional covariates in regression models resulted in slightly fewer included countries (n = 158). As this study did not involve people, medical records, human tissues, or any other personally identifiable information, it did not require approval from an Institutional Review Board

**Table 1:** Correlation matrix showing Pearson’s correlation coefficients for bivariate comparisons of all variables included in regression analyses.

	Age-Adjusted Leukemia Incidence per 100,000 (Males, 2012)	Age-Adjusted Leukemia Incidence per 100,000 (Females, 2012)	Cloud Cover-Adjusted Ultraviolet B Irradiation (Watts/m^2^)	Average Elevation (meters)	Male Life Expectancy (years, 2012)	Female Life Expectancy (years, 2012)
Age-Adjusted Leukemia Incidence per 100,000 (Males, 2012)	1	0.891*	-0.641*	-0.173*	0.730*	0.684*
Age-Adjusted Leukemia Incidence per 100,000 (Females, 2012)	0.891*	1	-0.567*	-0.180*	0.707*	0.660*
Cloud Cover-Adjusted Ultraviolet B Irradiation (Watts/m^2^)	-0.641*	-0.567*	1	0.166*	-0.695*	-0.637*
Average Elevation (meters)	-0.173*	-0.180*	0.166*	1	-0.1351	-0.1128
Male Life Expectancy (years, 2012)	0.730*	0.707*	-0.695*	-0.1351	1	0.965*
Female Male Life Expectancy (years, 2012)	0.684*	0.660*	-0.637*	-0.1128	0.965*	1

*p < 0.05

## Results

Leukemia incidence rates for 172 countries were inversely associated with cloud-adjusted UVB irradiance in males (*p* < 0.01) and females (*p* < 0.01) in both hemispheres (Fig 1, Fig 2). There were few departures from the trend line, which was parabolic when plotted with the equator at the center of the display, northern hemisphere countries on the right side, and southern hemisphere countries on the left. The association persisted in males (*p* < 0.01) and females (*p* < 0.01) after controlling for life elevation and sex-specific life expectancy (Table 2, Table 3)

**Table 2:** Multiple linear regression model for age-standardized leukemia incidence rates according to cloud-adjusted UVB irradiance, males, 158 countries, 2012 (*R* ^2^ = 0.62; *p* < 0.0001).

Covariate	Regression coefficient	Standard error	*t*	*p*
Solar UVB irradiance, Watts/m^2^	-0.18	0.053	-3.37	<0.01
Elevation, meters	-0.0025	0.00026	-0.96	0.34
Male life expectancy, years	0.18	0.021	8.29	<0.01
Intercept	-5.74	1.94	-2.96	<0.01

**Table 3:** Multiple linear regression model for age-standardized leukemia incidence rates according to cloud-adjusted UVB irradiance, females, 158 countries, 2012 (*R* ^2^ = 0.58; *p* < 0.0001).

Covariate	Regression coefficient	Standard error	*t*	*p*
Solar UVB irradiance, Watts/m^2^	-0.11	0.034	-3.10	<0.01
Elevation, meters	-0.00033	0.00018	-1.78	0.07
Female life expectancy, years	0.12	0.015	8.56	<0.01
Intercept	-3.83	1.26	-3.04	<0.01

## Discussion

The results of this study show that populations with higher exposure to cloud cover-adjusted UVB irradiance experienced lower incidence rates of leukemia. This is a novel advance in that leukemia incidence has not previously been compared to UVB irradiance after adjustment for cloud cover using satellite data. Use of cloud-adjusted UVB irradiance provided compliance of the incidence rates to the parabolic curve.

Importantly, these results suggest that increased levels of UVB irradiance and vitamin D may help prevent development of leukemia. Populations farther away from the equator will, on average, be exposed to solar energy that has traveled farther through the Earth’s atmosphere, thereby lowering the amount of UVB available to the skin [19].

Wavelengths between 270 and 300 nm allow for the creation of vitamin D in that they are absorbed by 7-dehydrocholesterol, a molecule found in the skin, which then undergoes photoconversion into vitamin D [20]. It then enters the bloodstream where it is metabolized to 25-hydroxyvitamin D in the liver, which then travels to the kidneys, where it is metabolized to 1,25-dihydroxyvitamin D. In regard to prevention of leukemias, this molecule acts on vitamin D receptors in bone marrow to produce e-cadherins [21], which make up adherens junctions that bridge intracellular actin cytoskeletons. These junctions help to tightly bind cells together, causing increased contact inhibition of cancer [9]. This works in part by phosphorylating certain membrane proteins, which are then translocated to the nucleus, where this process results in transcription of G_1_-phase inhibitors [22], which control mitosis and therefore may help to prevent the uncontrolled proliferation of white blood cells that is characteristic of leukemia. Several studies specifically conducted on HL-60 leukemia cells indicate that 1,25-dihydroxyvitamin D may mechanistically exert its effect through a cytosolic receptor on the MEK pathway [23] in order to upregulate G_1_ phase inhibitors like p27^Kip1^ and p21^Cip1^ [24], which are findings that support the likelihood of 1,25-dihydroxyvitamin D’s influence on leukemia prevention by regulating transcription in favor of G_1_-phase inhibition.

Several epidemiological studies have suggested that inadequate levels of vitamin D may be a risk factor for the development of leukemia [3–8]. In the Harvard Health Professionals Follow-Up Study, it was discovered that a 25 nmol/L increase in modeled 25-hydroxyvitamin D, the primary metabolite of vitamin D, was associated with a 66% decrease in risk of leukemia [25].

It was possible that other risk factors for leukemia might have confounded the relationship between leukemia prevention and UVB exposure. For this reason, elevation and life expectancy were included in the regression models. Data were not available by country on population exposures to ionizing radiation or organic solvents, so these could not be included as covariates.

An individual at higher elevation may be slightly more exposed to UVB irradiance. These wavelengths, which are more sensitive and less abundant than UVA wavelengths, would have to travel a shorter distance to reach an individual at higher elevation. While this covariate itself was not significantly associated with sex-specific leukemia incidence in the regression models, it was nonetheless included in an attempt to correct for possible confounding.

Groups of longer-living individuals are generally more likely to have greater exposure to environmental and behavioral factors that promote unregulated cellular proliferation. Therefore, this covariate broadly captures variations in leukemia-promoting environments and behaviors that could not be exhaustively included in the model. Furthermore, it has been documented that the skin tissue of older individuals has less 7-dehydrocholesterol, the molecule that converts to vitamin D upon interaction with UVB irradiance [20]. As a result of these qualities, the addition of this covariate adjusts for confounding of the relationship between UVB exposure and leukemia incidence.

Vitamin D replacement policies, such as fortification in food and discouragement of complete sun avoidance, may have the potential to lower leukemia incidence. These policies have been pursued in certain Nordic countries [26], which have relatively high rates of several cancers and relatively low levels of UVB irradiation, but thorough documentation has not been published as to whether these policies have had sufficiently broad reach or whether levels of fortification are adequate. It is worthwhile to note that vitamin D replacement policies could prudently target at-risk groups, especially the elderly, who have much higher rates of several cancers [27] and have relatively lower levels of 7-dehydroxyvitamin D [20].

### Strengths

The data source includes hundreds of thousands of cases from a large number of countries. Statistical analyses were used to account for the key confounders of elevation and life expectancy. This study provides ecological evidence that supports prior findings among individuals and tissue samples. This is consistent with ecological studies on colorectal [28] and breast cancers [29], where the association between vitamin D deficiency and cancer had also been established in many studies of individuals, including major cohort studies [30] and a randomized controlled clinical trial [31].

### Weaknesses

These analyses were not able to control for all confounders that may account for differences in leukemia risk between countries. Some of these confounders may be very influential on risk for leukemia. Furthermore, while this study did not suffer from bias derived from the collection of data, it is possible that the data source somewhat suffers from reporting bias, as population-based cancer registries in lower-income areas tend to cover lower proportions of inhabitants [32].

Our analyses are subject to the possibility of an ecological fallacy, so these findings may not be generalizable to individuals. It is possible that the effect size for the association between cloud cover-adjusted UVB and sex-specific, age-standardized leukemia incidence may be somewhat exaggerated due to the use of wintertime exposure, which represents the minimum amount of UVB irradiance across seasons. Also, grouping by subtypes of leukemia was not available from the GLOBOCAN database, so we were not able to distinguish between the different subtypes of leukemia. If the findings were null, we would be concerned that grouping of subtypes of leukemia could have masked some of the true associations. Since we found the associations reported here despite grouping of subtypes, this is less of a concern.

## Conclusions

In conclusion, this study showed that leukemia’s association with UVB exposure appeared similar to those seen for breast and colorectal cancers. Skin photosynthesis accounts for a large proportion of 25(OH)D concentration [33]. As a result, the inverse association between cloud–adjusted solar UVB exposure and incidence rates is likely to be mediated by circulating 25(OH)D, which is highly dependent on solar UVB irradiance. Also, clinical studies have shown similar associations in individuals as those seen in this report between countries [7, 8].

This study revealed a strikingly large difference in country-level leukemia risk when comparing equatorial countries to high-latitude countries. Studies comparing large regions, such as countries or subnational entities, may be more likely find greater differences in disease risk, given that the distinction between two large regions may be more likely to encompass widely different cultural practices and norms, such as those pertaining to diet, the components of which broadly influence risk for chronic diseases. While regression models in this study included life expectancy in order to control for this source of confounding, studies among individuals should be conducted in order to definitively determine whether differences in leukemia risk according to vitamin D status are consistent with those presented here with country-level UVB irradiance.

## Supporting Information

S1 Fig Rates of age-standardized leukemia incidence (cases per 100,000) in both sexes according to cloud cover-adjusted UVB irradiance (W/m^2^) with countries weighted according to total population, 172 countries, 2012. (TIF)

S2 Fig World map of leukemia incidence in both sexes (per 100,000 people), represented by graduated symbols, overlaid upon cloud cover-adjusted UVB irradiance (Watts/m^2^), represented by a choropleth gradient, 172 countries, 2012. (TIF)

S1 Table Country-level data used in creating graphs and regression models. (XLS)
