# Supplementary figures and images for: Low Cloud Cover-Adjusted Ultraviolet B Irradiance Is Associated with High Incidence Rates of Leukemia: Study of 172 Countries
Source: PLoS One. 2015 Dec 4;10(12):e0144308. doi: 10.1371/journal.pone.0144308 (PMC4670097; doi:10.1371/journal.pone.0144308)

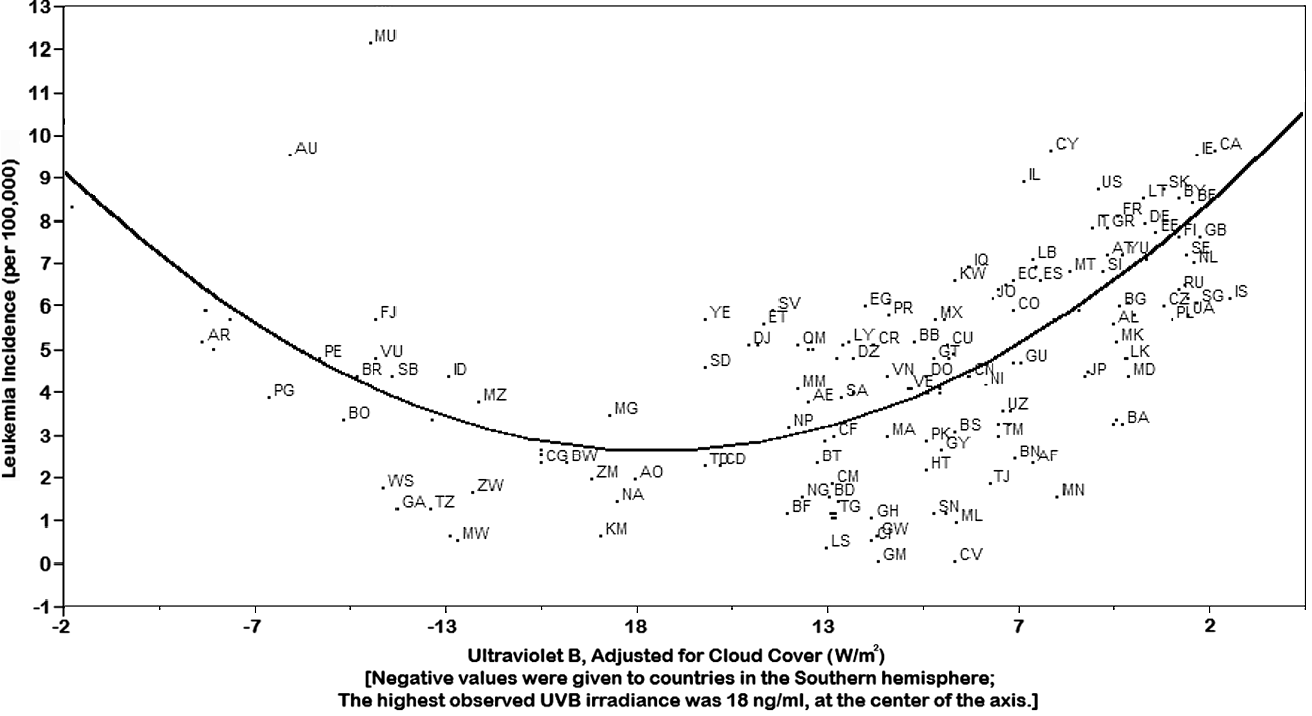

Supplement: S1 Fig — (TIF) [file pone.0144308.s001.tif]

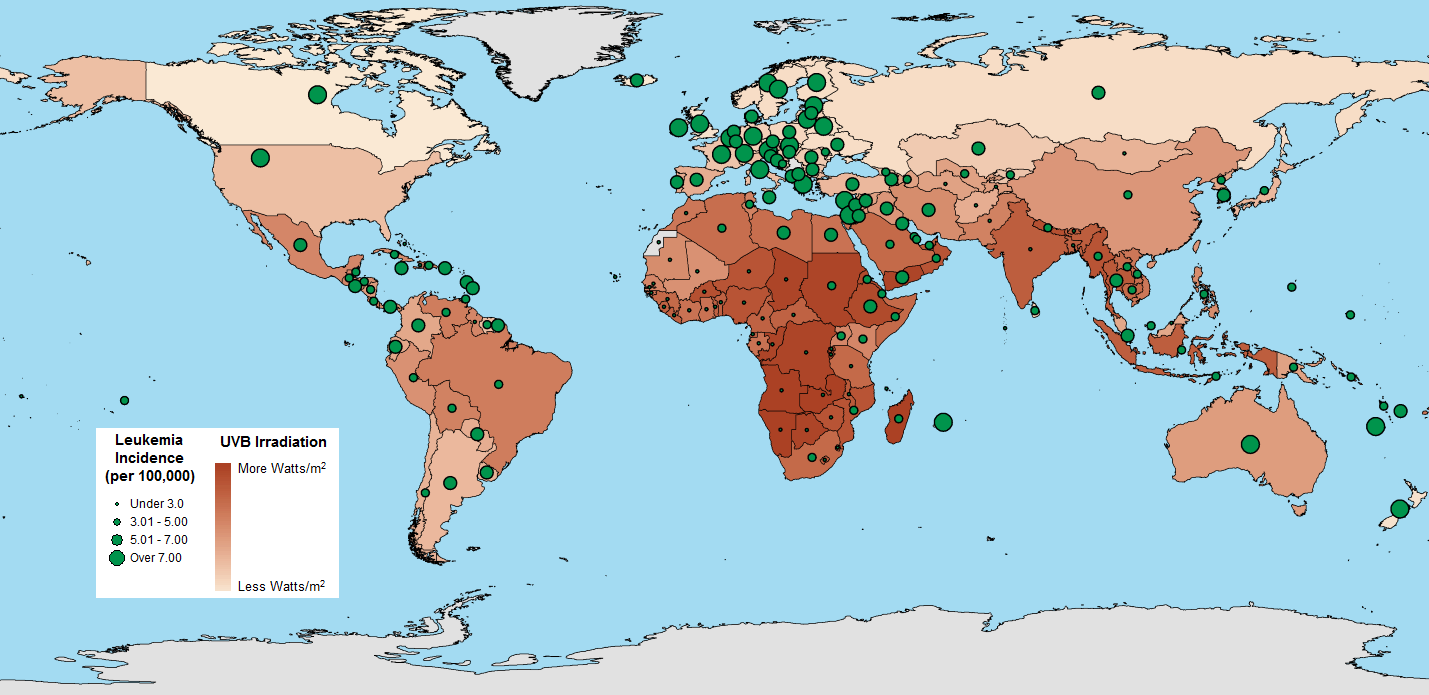

Supplement: S2 Fig — (TIF) [file pone.0144308.s002.tif]
